# Molecular mechanism of anionic stabilizer for telomere G-quadruplex

**DOI:** 10.52601/bpr.2022.220039

**Published:** 2022-08-31

**Authors:** Zhiguo Wang, Jianfeng Li, Jun Liu, Lihui Wang, Yanhua Lu, Jun-Ping Liu

**Affiliations:** 1 Institute of Ageing Research, School of Basic Medical Sciences, Hangzhou Normal University, Hangzhou 311121, China; 2 Department of Immunology, Central Eastern Clinical School, Monash University, Melbourne, Vitoria 3004, Australia; 3 Hudson Institute of Medical Research, Clayton, Victoria 3168, Australia; 4 Department of Molecular and Translational Science, Monash University, Clayton, Victoria 3168, Australia

**Keywords:** Telomere G-quadruplex, Anionic stabilizer, Selective mechanism, Molecular dynamics, MM/GBSA

## Abstract

Telomere DNA assumes a high-order G-quadruplex (G4) structure, stabilization of which prevents telomere lengthening by telomerase in cancer. Through applying combined molecular simulation methods, an investigation on the selective binding mechanism of anionic phthalocyanine 3,4ʹ,4ʹʹ,4ʹʹʹ-tetrasulfonic acid (APC) and human hybrid (3 + 1) G4s was firstly performed at the atomic level. Compared to the groove binding mode of APC and the hybrid type I (hybrid-I) telomere G4, APC preferred to bind to the hybrid type II (hybrid-II) telomere G4 via end-stacking interactions, which showed much more favorable binding free energies. Analyses of the non-covalent interaction and binding free energy decomposition revealed a decisive role of van der Waals interaction in the binding of APC and telomere hybrid G4s. And the binding of APC and hybrid-II G4 that showed the highest binding affinity adopted the end-stacking binding mode to form the most extensive van der Waals interactions. These findings add new knowledge to the design of selective stabilizers targeting telomere G4 in cancer.

## INTRODUCTION

As a non-canonical secondary structure of nucleic acids, G4 mostly located at the DNA and RNA guanine-rich regions such as telomeres, proto-oncogene promoters, and mRNA untranslated regions (Christiansen* et al.* 1994; Collie* et al.* 2010; Huppert and Balasubramanian 2005; Seenisamy* et al.* 2004; Siddiqui-Jain* et al.* 2002). Human telomeres are composed of a double-stranded region (several kilo-base pairs) and a single-stranded G-rich 3′ overhang (100–200 bases with the sequence of ([T_2_AG_3_]_n_)) (Bochman* et al.* 2012). Under physiological conditions, telomere G-rich overhang tends to fold into intramolecular G4 structures whose stabilization may inhibit telomerase that is required in 80%–95% of all malignant tumors (Mengual Gomez 2016; Phan 2010; Wu and Brosh 2010). Therefore, telomere G4 has been regarded as a potential target for cancer therapy, and stabilizer development targeting telomere G4 has attracted considerable interest (Balasubramanian* et al.* 2011; Onel* et al.* 2014).

Telomere G4s that comprise G-tetrads, TTA linking strands, central alkali metal ions, and capping ends, are polymorphic in strand orientations and glycosidic conformations (*anti* and *syn*) and present parallel, antiparallel, basket, and hybrid conformations (Attila* et al.* 2006; Dai* et al.* 2007; Lim* et al.* 2009; Parkinson* et al.* 2002; Phan and Patel 2003; Phan* et al.* 2006, 2007). Currently, a variety of telomere G4 stabilizers have been identified, with most of which sharing two structural features in common: a π-conjugated framework and a net cation charge (Yan* et al.* 2013). The conjugated framework of the stabilizer facilitates its π–π stacking interaction with the coplanar G-tetrad layers of G4, meanwhile, the cation charge increases binding affinity via electrostatic interaction with the negatively charged phosphodiester backbones of G4. However, the cation charge of the stabilizer causes a low selectivity for G4 over the double-stranded DNA (dsDNA) due to the non-directionality nature of electrostatic interaction. For instance, G4 stabilizer TMPyP4 which has four positively charged substituents loses its telomerase inhibitory activity under excessive dsDNA conditions (Martino* et al.* 2009; Ruan* et al.* 2016). In contrast, neutral stabilizer telomestatin binds to telomere G4 with high selectivity, but low solubility limits its pharmacological effect (Kim* et al.* 2002; Sumi* et al.* 2004).

**Figure 1:**
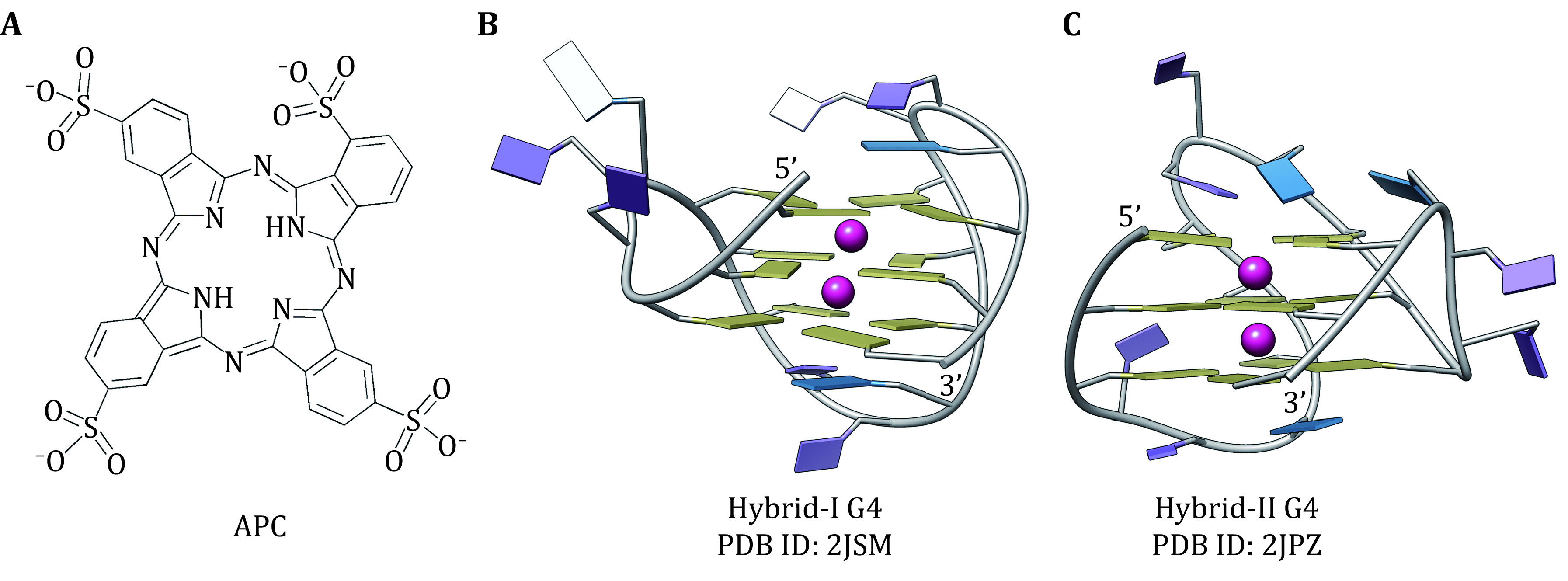
Structure of the negatively charged stabilizer APC (**A**), and the truncated structures of telomere hybrid-I G4 (**B**) and hybrid-II G4 (**C**). The bases of adenines, thymines, and guanines are colored in blue, purple, and khaki, respectively. The central potassium ions are shown as red spheres

Surprisingly, the negatively charged phthalocyanine APC (Fig. 1A) was found to bind to telomere hybrid G4s with advanced selectivity and good solubility, causing an efficient inhibition of telomerase activity even in the presence of excessive dsDNA (Yaku* et al.* 2010). However, some key information including the specific type of target hybrid G4, the binding mode of APC, and the selective binding mechanism remain to be learned.

Developing of G4 stabilizers that target a specific kind of G4 is rather challenging. Due to different loop arrangements and structural conformations (Fig. 1B and 1C), hybrid-I and hybrid-II G4s probably favor non-consensus binding modes and present varied binding affinities in binding with APC. Thus, elucidating their binding characteristics at the atomic level would provide valuable information for the design of selective stabilizers targeting telomere G4. In this study, we aimed to characterize the selective binding mechanism of APC to the hybrid telomere G4 through long time-scale molecular dynamics (MD) simulations, and to identify the decisive factor in the selective binding by performing non-covalent interaction (NCI) and binding free energy decomposition analyses.

## RESULTS

### Structure feature of telomere hybrid G4s

**Figure 2:**
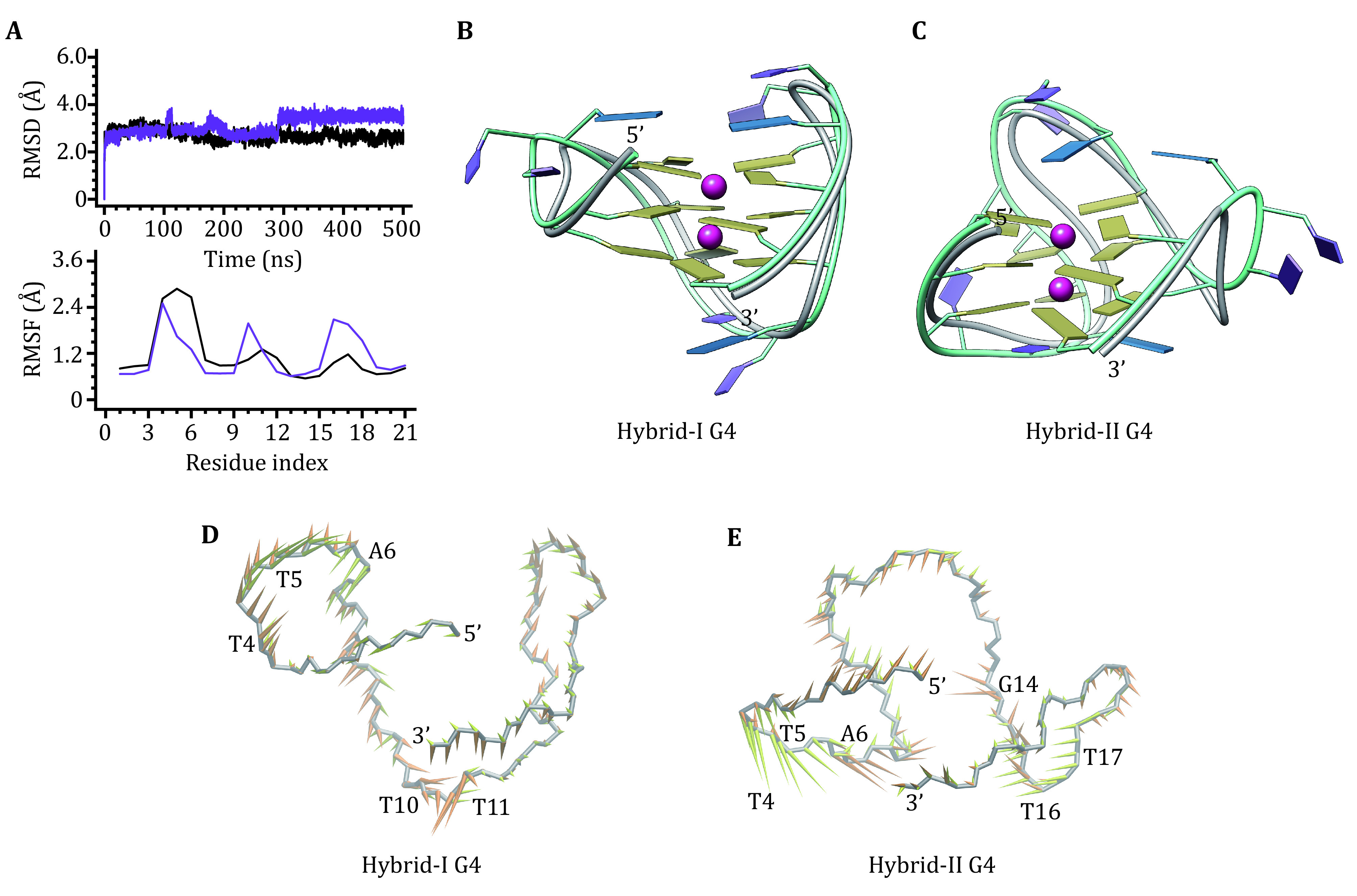
Characteristics of the apo hybrid-I and hybrid-II G4s. **A** RMSDs and RMSFs of telomere hybrid-I (black) and hybrid-II (purple) G4s. **B**, **C** MD-equilibrated structures of hybrid-I G4 and hybrid-II G4. For comparison, the initial NMR structures are shown as grey ribbons. **D**, **E** Porcupine plots of the first (yellow) and the second (orange) eigenvectors of hybrid-I G4 and hybrid-II G4

To characterize their structural feature, both telomere hybrid-I and hybrid-II G4s were subjected to MD simulation with a time scale of 500 ns. The root-mean-square deviation (RMSD) curve of hybrid-I G4 showed no drastic fluctuations, indicating marginal variations in the structural conformation. For the hybrid-II G4, the sudden increase in RMSD curve at about 292 ns mainly corresponded to the conformational alternation of the second edge-wise and the double-chain-reversal loops (Fig. 2A). Both root-mean-square fluctuation (RMSF) profiles shared a common feature, *i*.*e*., the relatively lower RMSF values of G-tetrad guanines and the higher RMSF values of loop nucleotides, especially the double-chain-reversal TTA loops, indicative of high structural flexibility of the linking strands (Fig. 2A). Structure alignment of the MD-equilibrated structures of hybrid-I/hybrid-II G4s and their respective NMR structures showed a good superposition at the G-tetrad regions and obvious deviations at the loop regions (Fig. 2B and 2C). In PCA analysis, eigenvalues of the first 30 eigenvectors demonstrated that 74.60% (56.00%) and 76.22% (59.41%) motions of hybrid-I and hybrid-II G4s can be specified by the first five (two) eigenvectors (supplementary Fig. S1a). Free energy landscape (FEL) maps whole alternative conformations of a molecular towards their corresponding energy as the Gibbs free energy. The FEL contours of the apo hybrid-I and hybrid-II G4s that were constructed based on the principal component 1 (PC1) and principal component 2 (PC2), showed diverse low-energy conformational states by separated clusters (supplementary Fig. S2a) and converged conformation by the single cluster, respectively (supplementary Fig. S2d). Porcupine plot identified that for the hybrid-I G4 the first and the second eigenvectors mainly corresponded to the motions of double-chain-reversal (T4, T5, and A6) and the first edge-wise (T10 and T11) loops (Fig. 2D). For the hybrid-II G4, the motions of the first edge-wise (T4 and T5) and the double-chain-reversal (T16 and T17) loops were indicated by the first eigenvector, and the motions of the first edge-wise loop (A6) and the bottom G-tetrad (G14) were indicated by the second eigenvector (Fig. 2E). The identified motion features agree well with the RMSF result and structural alignment.

### Binding modes of APC to telomere hybrid G4s

**Figure 3:**
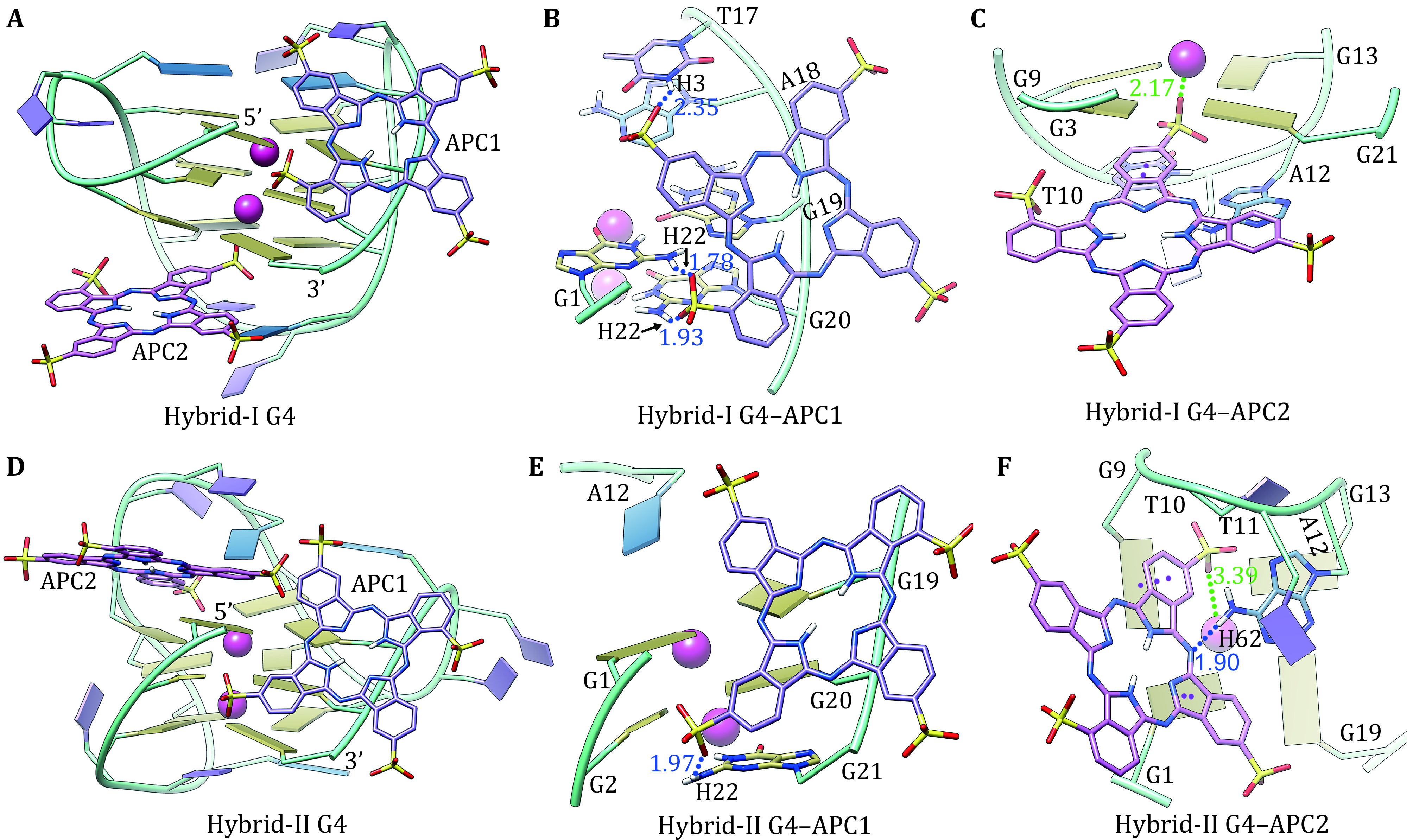
Binding conformations of APC with telomere hybrid-I and hybrid-II G4s. APCs bound to the groove and the exposed G-tetrad are denoted as APC1 and APC2, respectively. **A** Binding conformation of APC and hybrid-I G4. **B** The groove binding of APC1 and hybrid-I G4. **C** The partial end-stacking binding of APC2 and hybrid-I G4. **D** Binding conformation of APC and hybrid-II G4. **E** The groove binding of APC1 and hybrid-II G4. **F** The partial end-stacking binding of APC2 and hybrid-II G4. The hydrogen bonds, electrostatic interactions, and π–π stacking interactions are denoted by blue, green, and purple dotted lines, respectively

Based on the creteria of RMSD value of 1.0 Å, the docked APC molecules were grouped, with the ones that showed the most negative binding energies in the main groups recognized as potent bioactive binding conformations (Wang and Liu 2017). For each of the MD-equilibrated hybrid G4s, molecular docking identified two distinct binding modes, *i*.*e*., the groove binding (APC1) and the end-stacking binding (APC2) (Fig. 3). In the groove binding mode, APC1 located in the medium-sized groove formed between 5ʹ- and 3ʹ-terminals of hybrid-I G4 (Fig. 3A and 3B). The intermolecular hydrogen bonds between the oxygen atoms of sulfonic groups of APC1 and the atoms of H22 (G1), H3 (T17), and H22 (G20) contributed most to the groove binding (Fig. 3B). In the end-stacking binding mode, through partially intercalating between T10 of the first edge-wise loop and G3 of the bottom G-tetrad, APC2 formed parallel-displaced π–π stacking interactions with hybrid-I G4 (Fig. 3C). In addition, the electrostatic interactions between the inserted sulfonic group of APC2 and the bottom K^+^ also contributed to the end-stacking binding (Fig. 3C).

In binding with hybrid-II G4 APC1 located in the same groove with one intermolecular hydrogen bond formed between the oxygen atom of sulfonic group of APC1 and the of H22 (G21) (Fig. 3D and 3E). Different from the partial end-stacking binding of APC2 and hybrid-I G4, APC2 stacked over the top G-tetrad of hybrid-II G4 (G1 and G9) to form π–π interactions and electrostatic interactions with the top K^+ ^ (Fig. 3F). In addition, an intermolecular hydrogen bond between APC2 and the atom of H62 (A12) was observed with a bond length of 3.39 Å (Fig. 3F).

### Dynamic features of the hybrid G4‒APC binding structures

**Figure 4:**
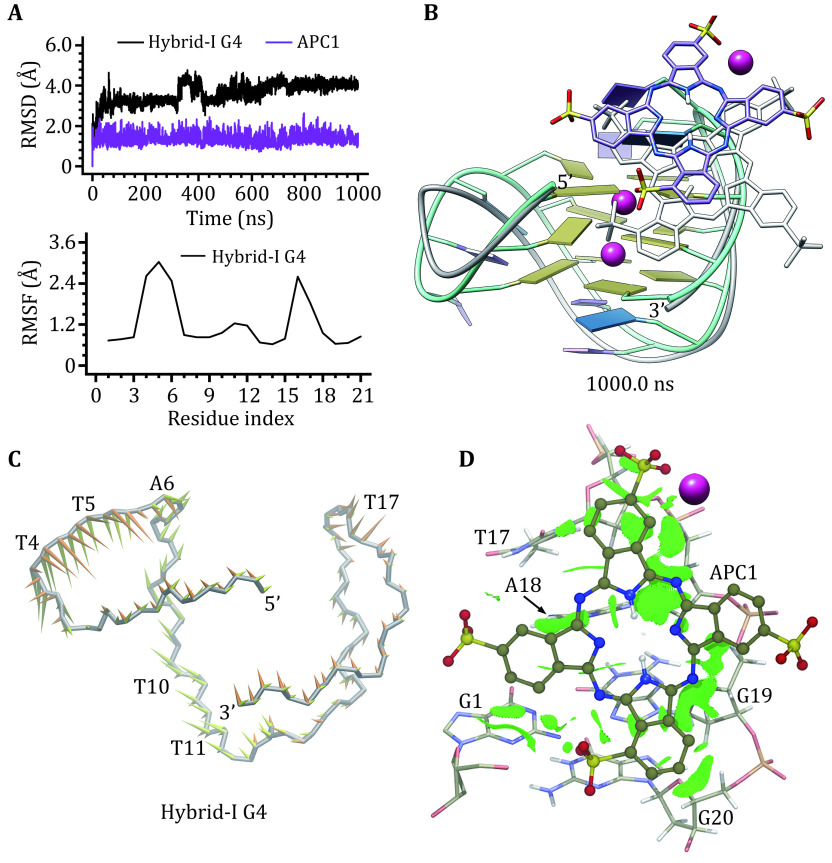
Binding characteristics of the hybrid-I G4‒APC1 complex. **A** RMSD and RMSF profiles. **B** MD-equilibrated binding conformation of hybrid-I G4 and APC1. For comparison, the initial structures are shown as grey ribbon (hybrid-I G4) and grey stick (APC1), respectively. **C** Porcupine plot of the first (yellow) and the second (orange) eigenvectors of hybrid-I G4. **D** NCI surface around the bound APC1 in the groove binding site of hybrid-I G4 (isovalue of 0.3 au)

In binding with hybrid-I G4 APC1 remained binding in the medium-sized groove. RMSD profiles of hybrid-I G4 and APC1 indicated equilibration of MD ensemble within 1000 ns (Fig. 4A). Visual inspection identified that the RMSD fluctuations of hybrid-I G4 mainly corresponded to the conformational alternations of the loop regions, which is in agreement with the RMSF profile and structural comparison (Fig. 4A and 4B). The smooth RMSD curve of APC1 was in accordance with its slight positional shift in the binding groove (Fig. 4A and 4B). PCA analysis found that about 39.98% of concerted motions of hybrid-I G4 can be specified by the first two eigenvectors (supplementary Fig. S1b). The FEL of the APC1 bound hybrid-I G4 showed separate cluster regions, indicating a transition between low-energy conformations (supplementary Fig. S2b). The first eigenvector mainly stood for the motions of the double-chain-reversal (T4, T5 and A6) and the first edge-wise (T10 and T11) loops, and the second eigenvector mainly stood for the motions of double-chain-reversal (T4 and T5) and the second edge-wise (T17) loops (Fig. 4C). The intermolecular hydrogen bonds observed in the docking conformation (Fig. 3B) were unstable with a hydrogen bond occupancy (HBO) of being less than 5% during MD simulation and disappeared in the final structure (Fig. 4B). Further analysis by NCIplot revealed that hybrid-I G4 mainly used nucleotides of G1, T17, A18, G19, and G20 to form van der Waals (vdW) interactions with the bound APC1. However, the vdW interactions were weak as indicated by the fragmented isosurface (Fig. 4D).

**Figure 5:**
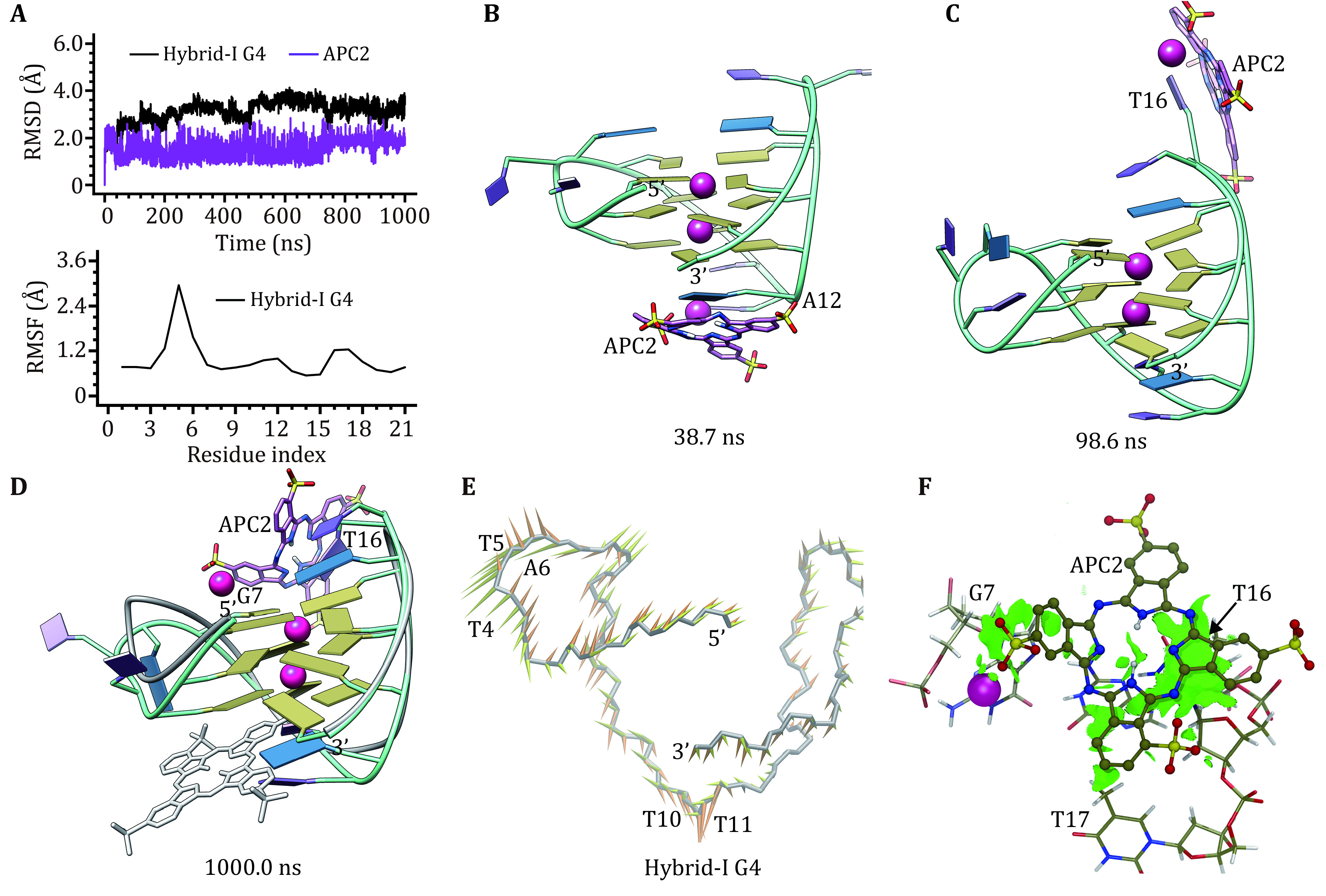
Binding characteristics of the hybrid-I G4‒APC2 complex. **A** RMSD and RMSF profiles. **B**, **C** Binding conformations at 38.7 and 98.6 ns, respectively. **D** MD-equilibrated binding conformation of hybrid-I G4 and APC2. For comparison, the initial structures are shown as grey ribbon (hybrid-I G4) and grey stick (APC2), respectively. **E** Porcupine plot of the first (yellow) and the second (orange) eigenvectors of hybrid-I G4. **F** NCI surface around the bound APC2 in the major groove binding site of hybrid-I G4 (isovalue of 0.3 au)

In binding with hybrid-I G4 APC2 changed to bind in the major groove from the initial end-stacking binding mode. Agreeing with the RMSF profile and structural comparison result, RMSD fluctuations of hybrid-I G4 were mainly caused by the conformational alternations of loops, especially the double-chain-reversal loop (Fig. 5A and 5D). The increased fluctuations of the APC2 RMSD curve were caused by the changes in binding location. APC2 firstly disassociated from the initial end-stacking binding and formed π–π stacking interaction with A12 at 38.7 ns (Fig. 5B), then it dissolved into the solvent environment and changed to stack to T16 at 98.6 ns (Fig. 5C), leading to the inward flip of T16, and finally, APC2 stabilized to bind in the major groove via π–π interactions with G7 and T16 in the MD-equilibrated structure (Fig. 5D). PCA analysis identified the first two eigenvectors accountable for 43.29% of concerted motions of hybrid-I G4 (supplementary Fig. S1b), with the first eigenvector mainly standing for the motions of the double-chain-reversal loop (T4, T5 and A6) and the second eigenvector mainly standing for the motions of the first edge-wise loop (T10 and T11) (Fig. 5E). The dispersed single cluster in the FEL indicated a conformational rearrangement of the APC2 bound hybrid-I G4 (supplementary Fig. S2c). NCI analysis showed that vdW interactions are mainly located between APC2 aromatic group and the bases of G7, T16, and T17 in hybrid-I G4 (Fig. 5F).

**Figure 6:**
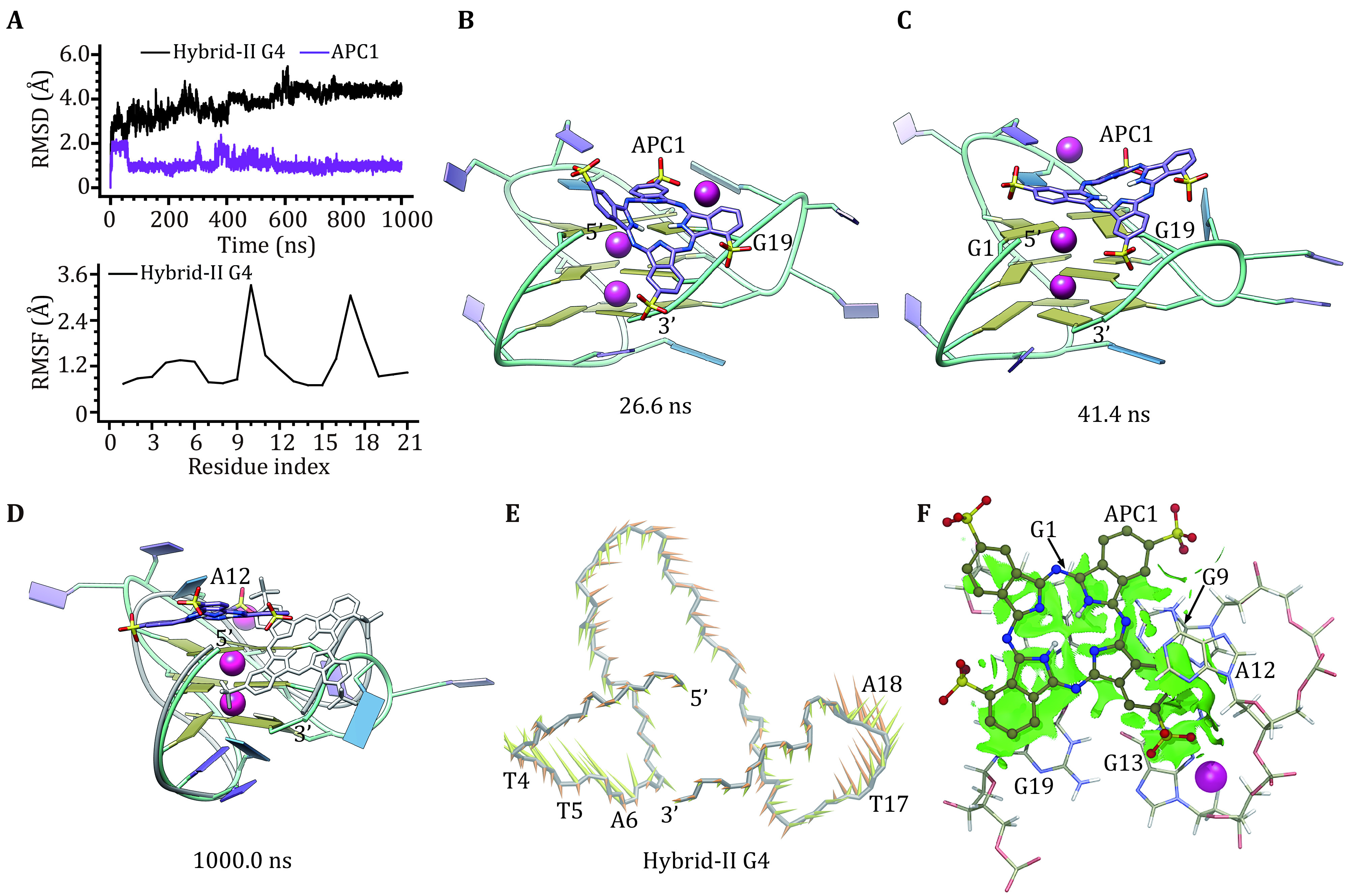
Binding characteristics of the hybrid-II G4‒APC1 binding complex. **A** RMSD and RMSF profiles. **B**, **C** Binding conformations at 26.6 and 41.4 ns, respectively. **D** MD-equilibrated binding conformation of hybrid-II G4 and APC1. For comparison, the initial structures are shown as grey ribbon (hybrid-II G4) and grey stick (APC1), respectively. **E** Porcupine plot of the first (yellow) and the second (orange) eigenvectors of hybrid-II G4. **F** NCI surface around the bound APC1 in the end-stacking binding site of hybrid-II G4 (isovalue of 0.3 au)

In binding with hybrid-II G4 APC1 changed to the end-stacking binding mode from initial groove binding. The high RMSD values of APC1 at the beginning stage corresponded to the changes in binding location (Fig. 6A‒6C), *i*.*e*., APC1 became disassociated from the groove binding and formed π–π stacking interaction with G19 at 26.6 ns (Fig. 6B), then APC1 formed increased π–π interactions with G1 and G19 at 41.4 ns (Fig. 6C), finally in the MD-equilibrated binding structure APC1 was completely stacked to the top G-tetrad. In comparison with the initial structure, the main conformational deviations of hybrid-II G4 occurred at the second edge-wise and the double-chain-reversal loops, which is in agreement with the RMSF profile (Fig. 6A and 6D). The conformational alternations of the APC1 bound hybrid-II G4 were indicated by the highly dispersed single cluster in the FEL (supplementary Fig. S2e). As identified by PCA analysis, the first two eigenvectors were accountable for 53.34% concerted motions of hybrid-II G4 (supplementary Fig. S1b). The first and the second eigenvector stood for the motions of the first edge-wise loop (T4, T5 and A6) and the double-chain-reversal loop (T17 and A18), respectively (Fig. 6E). As indicated by the green isosurface, NCI analysis presented a much more extensive van der Waals interactions between hybrid-II G4 (the top G-tetrad and A12) and APC1 compared to the hybrid-I G4−APC1/APC2 binding complexes (Fig. 6F), indicating a promoted binding affinity.

**Figure 7:**
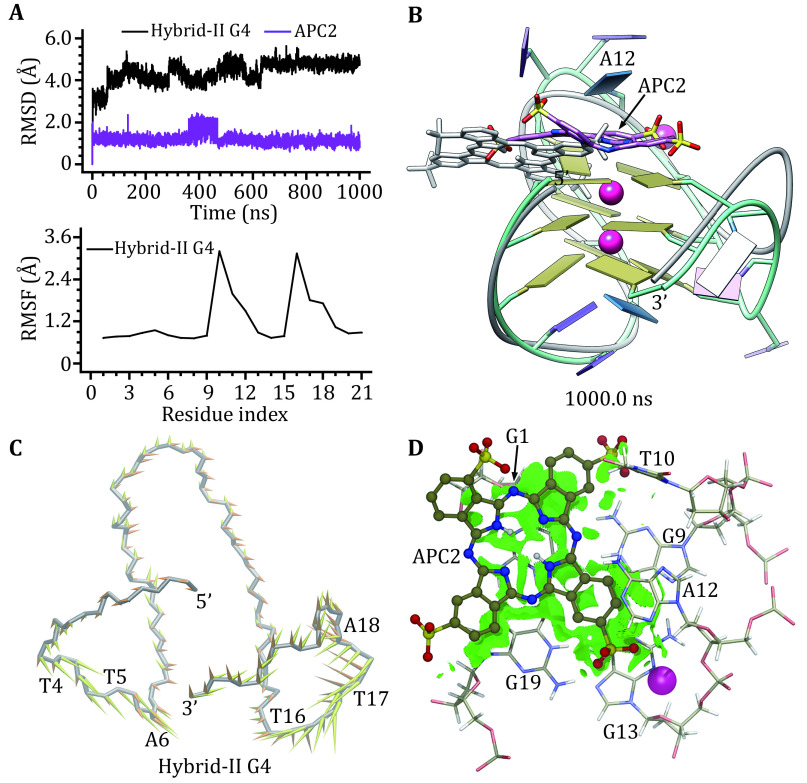
Binding characteristics of the hybrid-II G4‒APC2 binding complex. **A** RMSD and RMSF profiles. **B** MD-equilibrated binding conformation of hybrid-II G4 and APC2. For comparison, the initial structures are shown as grey ribbon (hybrid-II G4) and grey stick (APC2), respectively. **C** Porcupine plot of the first (yellow) and the second (orange) eigenvectors of hybrid-II G4. **D** NCI surface around the bound APC2 in the end-stacking binding site of hybrid-II G4 (isovalue of 0.3 au)

In binding with hybrid-II G4 APC2 remained the end-stacking binding mode. Except for the sudden increase between 361.9 ns and 469.2 ns that corresponded to the binding position shift of APC2, the RMSD curves showed no drastic fluctuations, indicating the stable binding of hybrid-II G4 and APC2 (Fig. 7A and 7B). Like APC1, APC2 stacked over the top G-tetrad in the equilibrated structure, and the shift in the binding position of APC2 facilitated extensive π–π stacking interactions with the top G-tetrad and the base of A12 (Fig. 7B). Both RMSF profile and structure comparison identified that conformational variations of hybrid-II G4 mainly occurred at the second edge-wise and the double-chain-reversal loops (Fig. 7A and 7B). The slightly diverged cluster in FEL indicated the conformational transition of the low-energy structures of hybrid-II G4 (Fig. S2F). As shown in the PCA analysis, 48.03% concerted motions of hybrid-I G4 were characterized by the first two eigenvectors (supplementary Fig. S1b), with the first eigenvector representing the motions of the first edge-wise (T4, T5 and A6) and the double-chain-reversal (T16, T17 and A18) loops and the second eigenvector representing the motions of the double-chain-reversal loop (T17 and A18), respectively (Fig. 7C). NCI analysis identified extensive vdW interactions between APC2 and the bases of hybrid-II G4 top G-tetrad and A12. In contrast to the fragmented isosurfaces of hybrid-I G4−APC1/APC2 binding complexes, the isosurfaces of hybrid-II G4−APC2 were as extensive as that of hybrid-II G4−APC1 (Fig. 7D).

### Stabilizing effect of APC to telomere hybrid G4s

Hoogsteen hydrogen bonds including O6∙∙∙H1−N1 and N7∙∙∙H21−N2 are formed between four guanine bases within each G-tetrad. It acts as one of the top contributing factors in the maintenance of G-tetrad layers, with the hydrogen bond status serving as a marker of G4 stability (Wang *et al*. 2018; Wang and Liu 2017). Analysis on hydrogen bond was performed under the criteria of bond length <3.5 Å and bond angle >120°. Parameters including HBO, hydrogen bond length, and angle were summarized in supplementary Table S1. For the apo hybrid-I G4, the minimal (average) HBOs of O6∙∙∙H1−N1 and N7∙∙∙H21−N2 were 90.49% (92.61%) and 98.76% (99.42%), respectively. The high HBO values indicated the structural stability of hybrid-I G4. For the apo hybrid-II G4, much lower minimal (average) HBOs, *i*.*e*., 78.41% (84.55%) for O6∙∙∙H1−N1 and 78.48% (89.00%) for N7∙∙∙H21−N2, were observed in the bottom G-tetrad. It should be noted that except for the O6∙∙∙H1−N1 in the central G-tetrad, all the averaged HBOs in hybrid-II G4 were lower than that in hybrid-I G4 (Table 1 and supplementary Table S1), indicating the less structural stability of hybrid-II G4.

**Table 1:** Characteristics of Hoogsteen hydrogen bonds within hybrid G4s

Model	Hydrogen bond^*a*^	Minimum			Maximum			Average	
		Ocpy^*b*^	Dist^*c*^		Ocpy	Dist		Ocpy	Dist
Hybrid-I G4	O6∙∙∙H1−N1^T^	99.54	2.94		99.89	2.93		99.74	2.93
	N7∙∙∙H21−N2^T^	99.23	2.97		99.82	3.02		99.60	2.99
	O6∙∙∙H1−N1^C^	90.49	3.16		95.90	3.10		92.61	3.15
	N7∙∙∙H21−N2^C^	98.76	3.00		99.86	2.94		99.42	2.98
	O6∙∙∙H1−N1^B^	99.04	3.00		99.94	2.93		99.65	2.97
	N7∙∙∙H21−N2^B^	99.64	2.97		99.92	2.96		99.78	2.97
Hybrid-I G4–APC1	O6∙∙∙H1−N1^T^	98.64	2.93		99.96	2.89		99.54	2.94
	N7∙∙∙H21−N2^T^	99.15	3.00		99.92	2.96		99.69	2.98
	O6∙∙∙H1−N1^C^	93.36	3.16		97.18	3.11		95.55	3.13
	N7∙∙∙H21−N2^C^	99.35	2.99		99.93	2.96		99.75	2.97
	O6∙∙∙H1−N1^B^	99.40	3.01		99.92	2.93		99.74	2.96
	N7∙∙∙H21−N2^B^	99.04	3.03		99.94	2.94		99.64	2.98
Hybrid-I G4–APC2	O6∙∙∙H1−N1^T^	99.68	2.94		99.80	2.92		99.74	2.93
	N7∙∙∙H21−N2^T^	99.01	2.99		99.92	2.99		99.43	3.00
	O6∙∙∙H1−N1^C^	90.75	3.17		94.54	3.13		92.71	3.16
	N7∙∙∙H21−N2^C^	99.21	2.98		99.77	2.96		99.60	2.98
	O6∙∙∙H1−N1^B^	98.91	2.98		99.91	2.93		99.40	2.98
	N7∙∙∙H21−N2^B^	99.70	2.97		99.91	2.96		99.77	2.96
Hybrid-II G4	O6∙∙∙H1−N1^T^	99.55	2.96		99.75	2.95		99.69	2.94
	N7∙∙∙H21−N2^T^	98.63	3.06		99.87	2.97		99.41	2.99
	O6∙∙∙H1−N1^C^	88.55	3.14		96.82	3.05		93.04	3.09
	N7∙∙∙H21−N2^C^	98.16	3.02		99.75	2.97		99.33	2.98
	O6∙∙∙H1−N1^B^	78.41	2.94		90.93	2.99		84.55	2.97
	N7∙∙∙H21−N2^B^	78.48	3.00		99.54	2.98		89.00	2.99
Hybrid-II G4–APC1	O6∙∙∙H1−N1^T^	99.93	2.90		99.97	2.90		99.95	2.90
	N7∙∙∙H21−N2^T^	96.05	3.01		99.79	3.02		98.77	3.01
	O6∙∙∙H1−N1^C^	90.90	3.18		97.13	3.07		94.43	3.12
	N7∙∙∙H21−N2^C^	99.48	2.98		99.83	2.97		99.68	2.98
	O6∙∙∙H1−N1^B^	99.31	2.93		99.73	2.96		99.50	2.95
	N7∙∙∙H21−N2^B^	99.38	2.96		99.71	2.99		99.54	2.98
Hybrid-II G4–APC2	O6∙∙∙H1−N1^T^	99.84	2.92		99.95	2.90		99.92	2.90
	N7∙∙∙H21−N2^T^	91.75	3.05		99.83	2.97		97.66	3.01
	O6∙∙∙H1−N1^C^	92.30	3.17		98.45	3.03		96.09	3.09
	N7∙∙∙H21−N2^C^	98.28	3.01		99.82	2.96		99.38	2.98
	O6∙∙∙H1−N1^B^	99.62	2.96		99.86	2.96		99.72	2.95
	N7∙∙∙H21−N2^B^	99.27	2.99		99.90	2.97		99.50	2.98
^*a*^ The subscripted words of T, C, and B indicate the hydrogen bonds located in the top, central, and bottom G-tetrads of hybrid G4s, respectively ^*b*^ Hydrogen bond occupancy during MD (%) ^*c*^ Time averaged hydrogen bond length (Å)									

The stabilizing effect of APC on telomere hybrid G4s was evaluated through the comparison of HBO values corresponding to the intramolecular Hoogsteen hydrogen bonds in the apo and the APC-bound hybrid G4s. For hybrid-I G4, binding with APC1 increased the minimal (average) HBOs of O6∙∙∙H1−N1 and N7∙∙∙H21−N2 to 93.36% (95.55%) and 99.35% (99.75%) from 90.49% (92.61%) and 98.76% (99.42%) (Table 1), showing an obvious stabilizing effect. Whereas APC2 can hardly improve the HBOs of Hoogsteen hydrogen bonds in hybrid-I G4. For the APC2-bound hybrid-I G4, the minimal (average) HBOs of O6∙∙∙H1−N1 and N7∙∙∙H21−N2 were 90.75% (92.71%) and 99.21% (99.60%) (Table 1), respectively, demonstrating a negligible stabilizing effect of APC2.

Through binding to hybrid-II G4, APC1 and APC2 greatly increased the minimal (average) HBOs of O6∙∙∙H1−N1 and N7∙∙∙H21−N2 to 99.31% (99.50%) and 99.38% (99.54%) and to 99.62% (99.72%) and 99.27% (99.50%), respectively (Table 1). In addition, the HBOs of O6∙∙∙H1−N1 and N7∙∙∙H21−N2 within the central G-tetrad were apparently improved by both APC1 and APC2 (Table 1). These results indicated that APC can effectively promote the structural stability of hybrid-II G4 through end-stacking binding.

### Characteristics of binding affinity

Binding affinities between APC and telomere hybrid G4s were evaluated by binding free energy calculations with the molecular mechanics/generalized Born surface area (MM/GBSA) approach, the results were summarized in Table 2. In contrast to the binding of cationic and neutral stabilizers (Wang *et al*. 2018), the electrostatic interaction and the polar solvation effect were found to make unfavorable and favorable contributions to the binding of APC and hybrid G4s, respectively (Table 2). Because of the fact that the unfavorable contributions coming from the electrostatic interactions were mostly compensated by the polar solvation effects and the entropy made unfavorable contributions with similar scales in all binding complexes, the vdW interactions actually played the decisive role in evaluating the overall binding free energies (Table 2). Compared to the binding with Hybrid-I G4, both APC1 and APC2 showed promoted binding free energies by 20 kcal/mol more negative in binding with Hybrid-II G4, indicating the preference of APC to Hybrid-II G4 through the end-stacking binding.

**Table 2:** Binding free energies between APC and telomere hybrid G4s

G4	Stabilizer	Energy component^*a*^					
		Δ*E*_ele_	Δ*E*_vdW_	Δ*G*_GB,sol_	Δ*G*_np,sol_	−*T*Δ*S*	Δ*G*_bind_
Hybrid-I G4	APC1	350.72	−42.02	−339.48	−4.33	24.00	−11.10
	APC2	352.79	−40.41	−342.06	−4.06	23.13	−10.62
Hybrid-II G4	APC1	358.50	−62.95	−345.29	−5.54	24.08	−31.20
	APC2	374.56	−66.74	−359.98	−5.88	25.16	−32.87
^*a*^ Energies are in kcal/mol							

**Figure 8:**
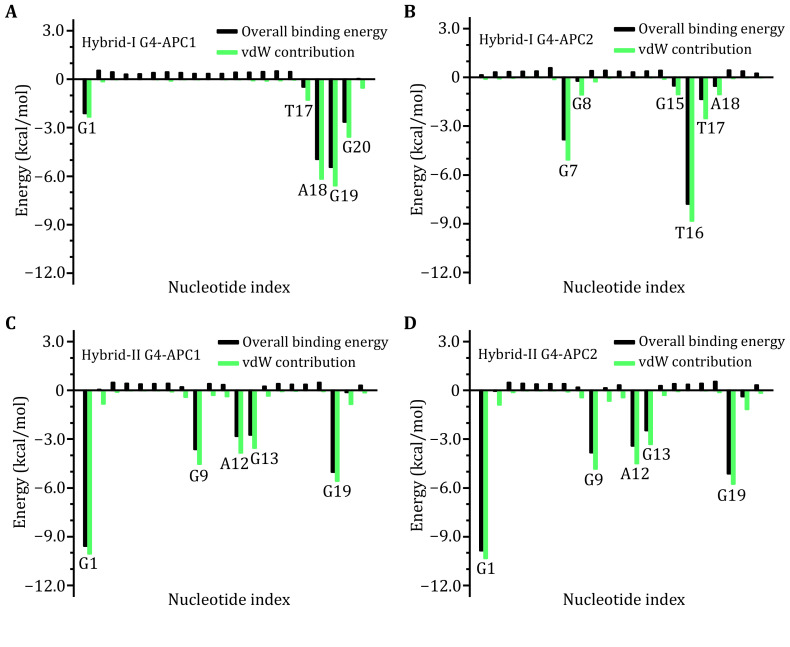
Per-residue decomposition of binding free energy. The van der Waals and overall contributions are represented by green and black columns, respectively. **A** Hybrid-I G4‒APC1. **B** Hybrid-I G4‒APC2. **C** Hybrid-II G4‒APC1. **D** Hybrid-II G4‒APC2

To identify the key residues that are accountable for the effective binding, per-residue decomposition of binding free energy was performed (Weng *et al*. 2019). As shown in Fig. 8, nucleotides G1, A18, G19, and G20 made the largest contributions to the binding of hybrid-I G4 and APC1 (Fig. 8A). For the binding of hybrid-I G4 and APC2, G7, T16, and T17 were the most contributing nucleotides (Fig. 8B). In binding with APC1 and APC2, same key nucleotide residues of hybrid-II G4 including A12 and the top G-tetrad bases (G1, G9, G13, and G19) and were identified (Fig. 8C and 8D). It should be noted that all the most contributing nucleotides formed considerable vdW interactions with the bound APC (Fig. 8). In addition, the identified key nucleotides were in good agreement with the NCI analysis (Figs. 4‒7), further confirming the pivotal role of van der Waals interaction in the bindings of APC and hybrid G4s.

## DISCUSSION

Our analyses demonstrated that the dual binding form between APC and each of hybrid-I and hybrid-II G4s finally converged to one specific binding mode, and APC under the groove and end-stacking binding modes showed a significant discrepancy in binding affinity. Alignment of the MD-equilibrated structures showed that APC1 and APC2 superimposed well in the binding site of hybrid-II G4 (supplementary Fig. S3), confirming the preference of APC for end-stacking binding mode. The numbers of environmental K^+ ^ within 3.5 Å of hybrid-I and hybrid-II G4s were analyzed and shown in supplementary Fig. S4. Interestingly, APC binding made the average numbers of environmental K^+ ^ surrounding hybrid-I and hybrid-II G4s increase by about 1 and 2, respectively (supplementary Fig. S4). The recruited K^+ ^ ions can form electrostatic interactions with APCand telomere G4s concurrently (Figs. 4‒7), which additionally contributed to the binding stability. Therefore, the binding free energy difference between groove binding and end-stacking binding should be greater since the MM/GBSA result did not include the contribution from the recruited K^+ ^. Combing all the obtained information, we elucidated that instead of binding to hybrid-I G4 through groove binding, APC preferred the energetically much more favorable end-stacking binding to hybrid-II G4. It is noteworthy that the current study provided a rational explanation for the high selectivity of APC to telomere G4 over dsDNA, given that groove binding is the most stable binding mode between dsDNA and ligand (Dalia* et al.* 2017; Machireddy* et al.* 2019; Nelson* et al.* 2007).

Many G4 stabilizers have been reported with binding properties. However, no G4 targeting drug has been approved due to selectivity issues *in vivo*. APC is the first negatively charged molecule that targets the telomere hybrid G4 with specificity. In the excess of dsDNA, APC remained inhibitory activity to telomerase, attenuating cancer cell proliferation (Yaku* et al.* 2010, 2012). The present work revealed that selective binding between APC and telomeric DNA G4 may be attributable to the electrostatic repulsions between the anionic sulfonic groups of APC and the anionic sugar-phosphate backbone of dsDNA. The findings here support the notion that metal coordinated derivatives of APC such as Cu(II)-APC and Ni(II)-APC show decreased IC_50_ values for the telomerase inhibition while maintaining good selectivity (Yaku* et al.* 2010).

In summary, molecular docking and MD simulations combined with PCA, NCI analysis, and MM/GBSA binding free energy decomposition illustrate the selective binding mechanism of APC to the hybrid-II telomere G4 at the atomic level. The current study provides new information on the design of the novel selective stabilizer targeting telomere G4.

## METHODS

### Data

The structures of telomere hybrid-I and hybrid-II G4s were retrieved from the PDB data bank with the IDs of 2JSM and 2JPZ, respectively (Dai* et al.* 2007; Phan* et al.* 2007). By deleting redundant terminal nucleotides both structures were modified to have the same sequence as the experiment model (5ʹ-GGGTTAGGGTTAGGGTTAGGG-3ʹ). Because of the importance of central metal ions in the structural stability of G4, potassium ions were added between the two adjacent G-tetrad layers (Fig. 1B and 1C). The structure of APC was constructed by using GaussView software, followed by optimization at the base level of DFT B3LYP/6-31G(d) (Wang *et al.* 2018; Wang and Liu 2017). The atomic partial charges of APC were calculated by applying the restricted electrostatic potential (RESP) method under the basis set of HF/6-31G(d) (Dalia* et al.* 2017; Wang *et al.* 2018). And the other force field parameters of APC were generated from the Generalized Amber Force field (GAFF) with the Antechamber module in AmberTools (Salomon-Ferrer* et al.* 2013).

### Molecular docking

Molecular docking calculations were performed using the AutoDock 4.2.6 software (Morris* et al.* 2009). The MD-equilibrated structures of the hybrid-I and hybrid-II G4s were set as receptors. APC was set as ligand with all the rotatable bonds set flexible. To define the binding region, a large cubic box comprising 100 × 100 × 100 girds with the grid spacing of 0.375 Å was used for the docking calculations, and the box center was set to overlap the geometry centers of hybrid-I and hybrid-II G4s. Each docking calculation comprised 150 genetic algorithm runs. Default values were used for all the other parameters.

### Molecular dynamics simulations

MD simulations were conducted by using the AMBER 12 software (Case* et al.* 2010). The apo hybrid G4s and the APC-bound hybrid G4 complexes were individually immersed into the center of a truncated octahedron box of TIP3P water molecules with a margin distance of 12.0 Å. Environmental potassium counterions were added to keep systems in electrical neutrality. The previously validated FF99SB force field combined with parmbsc1 and χ_OL3 + OL15_ modifications were applied for G4s (Machireddy* et al.* 2017, 2019). Standard Amber parameter (radius 2.658 Å and well depth 0.00328 kJ/mol) and calibrated parameter (radius 1.705 Å and well depth 0.1936829 kJ/mol) were used for the environmental K^+ ^ and the ones located between G-tetrad layers, respectively (Joung and Cheatham III 2008; Wang and Liu 2017). All MD simulations were conducted by following our previous reports (Wang *et al.* 2018, 2022; Wang and Liu 2017). Since simulations with insufficient time-scale were found limited in obtaining meaningful results in the MD studies of G4 (Islam* et al.* 2016), long time scales were applied to make sure that all models achieved equilibrations.

### Principal components analysis (PCA)

PCA filters essential degrees of freedom from a variety of local fluctuations in MD (Amadei* et al.* 1993). PCA analysis was carried out with VMD software by using the method of Interactive Essential Dynamics (IED) (Humphrey *et al.* 1996; Mongan 2004). The CPPTRAJ module in AmterTools16 was applied for hybrid G4 backbone atoms in PCA calculations. The graphical summary of motions along the first two eigenvectors was shown in the porcupine plot.

### Non-covalent interactions

NCIplot calculations were carried out with a step size of 0.10 to visualize the interacting regions between G4 and APC (Contreras-García* et al.* 2011). The reduced gradients were rendered as an isosurface in VMD (Humphrey *et al*. 1996), using an isovalue of 0.3 au.

### Binding free energy calculations

Since generalized Born (GB) models showed the ability to make good predictions on the hydration free energy of charged molecules (Machireddy* et al.* 2019; Wang* et al.* 2019), the binding free energies (Δ*G*_bind_) between hybrid G4s and APC were evaluated with the MM/GBSA approach (Kollman* et al.* 2000):

1 $ \Delta G_{{\rm{bind}}} = G_{{\rm{complex}}} – (G_{{\rm{G}}4} + G_{{\rm{APC}}})  $

2 $ \Delta G_{{\rm{bind}}} = \Delta H - T\Delta S \approx  \Delta E_{{\rm{MM}}} + \Delta G_{{\rm{solv}}} - T\Delta S $

3 $ \Delta E_{{\rm{MM}}} = \Delta E_{{\rm{int}}} + \Delta E_{{\rm{vdW}}} + \Delta E_{{\rm{ele}}} $

4 $ \Delta G_{{\rm{solv}}} = \Delta G_{{\rm{GB}}} + \Delta G_{{\rm{SA}}      } $

where *E*_MM_ is the interaction energy in the gas phase comprising internal strain energy (*E*_int_), van der Waals energy (*E*_vdW_), and electrostatic energy (*E*_ele_). *G*_solv_ is the solvation free energy, including the contributions from a polar part (*G*_GB_) and a nonpolar part (*G*_SA_). Δ*E*_int_ that comprises bond, angel, and dihedral energies would be cancelled as we used a single trajectory approach to reduce the noise. Δ*G*_GB_ was estimated using the GB model with the interior and exterior dielectric constants set to 4 and 80, respectively (Wang *et al*. 2019). Δ*G*_SA_ was estimated using the LCPO algorithm: Δ*G*_SA_ = *γ*Δ*SASA* + *β*, where *γ* and *β* were set to 0.0072 and 0, respectively (Weiser* et al.* 1999). *T*Δ*S* that represents entropy contribution was estimated through normal mode analysis (NMA) by using NMODE module (Sun *et al*. 2018). 500 snapshots were evenly extracted from the last 200 ns trajectories for the calculations of Δ*E*_vdW_, Δ*E*_ele_, Δ*G*_GB_, and Δ*G*_SA_. Due to the expensive computational cost of NMA, only 100 snapshots that were evenly extracted from the last 200 ns trajectories were used for the entropy calculations (*T*Δ*S*).

## Abbreviations

**Table:** 

G4	G-quadruplex
APC	Anionic phthalocyanine 3,4ʹ,4ʹʹ,4ʹʹʹ-tetrasulfonic acid
dsDNA	Double-stranded DNA
MD	Molecular dynamics
NCI	Non-covalent interaction
RMSD	Root-mean-square-deviation
RMSF	Root-mean-square-fluctuation
HBO	Hydrogen bond occupation
MM/GBSA	Molecular mechanics/generalized Born surface area
GB	Generalized Born
NMA	Normal mode analysis surface area

## Conflict of interest

Zhiguo Wang, Jianfeng Li, Jun Liu, Lihui Wang, Yanhua Lu and Jun-Ping Liu declare that they have no conflict of interest.
